# Effect of Storage Conditions on the Volatilome, Biochemical Composition and Quality of Golden Delicious and Red Delicious Apple (*Malus domestica*) Varieties

**DOI:** 10.3390/molecules29132954

**Published:** 2024-06-21

**Authors:** Cláudio Ferreira, Carlos Ribeiro, Fernando M. Nunes

**Affiliations:** 1 Food and Wine Chemistry Laboratory, Chemistry Research Center—Vila Real (CQ-VR), School of Life and Environmental Sciences, University of Trás-os-Montes and Alto Douro (UTAD), 5000-801 Vila Real, Portugal; claudio18ferreira@gmail.com; 2 Research and Technology Center for Agro-Environmental and Biological Sciences (CITAB), University of Trás-os-Montes and Alto Douro (UTAD), 5000-801 Vila Real, Portugal; cribeiro@utad.pt

**Keywords:** ‘Golden Delicious’, ‘Red Delicious’, firmness, volatilome, EE-α-farnesene, sugars, malic acid, 1-methylcyclopropene, controlled atmosphere storage, normal atmosphere storage

## Abstract

The effects of normal (NA) and controlled atmosphere (CA) storage and postharvest treatment with 1-methylcyclopropene (1-MCP) before CA storage for 5 months on the volatilome, biochemical composition and quality of ‘Golden Delicious’ (GD) and ‘Red Delicious’ (RD) apples were studied. Apples stored under NA and CA maintained and 1-MCP treatment increased firmness in both cultivars. NA storage resulted in a decrease of glucose, sucrose and fructose levels in both cultivars. When compared to CA storage, 1-MCP treatment caused a more significant decrease in sucrose levels and an increase in glucose levels. Additionally, 1-MCP-treated apples exhibited a significant decrease in malic acid content for both cultivars. All storage conditions led to significant changes in the abundance and composition of the volatilome in both cultivars. GD and RD apples responded differently to 1-MCP treatment compared to CA storage; higher abundance of hexanoate esters and (*E*,*E*)-α-farnesene was observed in RD apples treated with 1-MCP. While 1-MCP was effective in reducing (*E*,*E*)-α-farnesene abundance in GD apples, its impact on RD apples was more limited. However, for both cultivars, all storage conditions resulted in lower levels of 2-methylbutyl acetate, butyl acetate and hexyl acetate. The effectiveness of 1-MCP is cultivar dependent, with GD showing better results than RD.

## 1. Introduction

In 2022, global apple production reached 95,835,965 tons, making it the third most produced fruit after bananas and watermelons. The leading producers were China (50%), the EU (13%), Turkey (5%) and the USA (5%) [1]. Although there are over 7500 known apple cultivars, only a few dominate the European market. The most common apple groups in Europe include ‘Golden Delicious’ (24%), ‘Gala’ (9%), ‘Idared’ (7%), ‘Jonagold’ (5%), ‘Red Jonaprince’ (5%), ‘Granny Smith’ (5%) and ‘Red Delicious’ (5%). Together, these varieties account for nearly 59% of the production area of dessert apples in Europe [2]. Apple quality is determined by factors such as appearance, firmness and flavour, as well as the absence of physiological and pathological disorders. As apples ripen, they soften. Firmness is the dominant factor in consumer acceptance, though sugar and acid content also contribute to the quality of specific cultivars [3]. Firmer apples are generally juicier, crisper and less mealy than softer ones [4]. Additionally, firmer fruits are better suited for long-term storage, facilitating shipping and ensuring timely availability in the fruit market. The flavour of apples is another important and distinctive feature, determined by both taste and aroma [5,6]. The taste of apples is primarily influenced by sugars and organic acids. The balance between these components plays a key role in determining the sweetness and sourness of apple [7]. On the other hand, the aroma of apples is composed of a complex mixture of volatile organic compounds (VOCs), specific to the species and often to the variety [6,8,9]. It is sensed through the interaction of these VOCs with human receptors. Apple aroma is influenced by over 370 VOCs [10,11,12,13] which are synthesized by the fruit during ripening and became more pronounced upon cellular disruption, such as biting and mastication [13,14]. Among these VOCs, only a minor set of chemical compounds, primarily esters, alcohols and aldehydes, can be distinctly perceived [12,15].

Apple aroma plays a crucial role in enhancing eating quality and consumer acceptance [16]. For instance, sensory evaluation of ‘Pink Lady’ apples revealed that 65% of consumers preferred fruit with higher emissions of aroma-active VOCs, despite these fruits displaying lower values for standard quality attributes [17]. Studies on consumer acceptability of seven ‘Fuji’ apple strains found a strong correlation between acceptability and certain volatile compounds emitted [18]. Treatment with 1-MCP was found to reduce flavour volatiles in ‘Gala’ apples, with consumer tests showing that they could distinguish between 1-MCP-treated and untreated fruit [19]. Similarly, in ‘Fuji’ apples, 1-MCP treatment resulted in the lowest aroma score [20].

Ethylene plays an important role in the ripening process of apples, stimulating the development of the fruit’s characteristic taste and causing changes in flesh texture. However, during storage, ethylene is undesirable, as it promotes further ethylene production in the fruit, accelerating ripening. It also leads to a decrease in organic acids and weakened cell connections, resulting in a softer flesh structure and deteriorated texture, which ultimately negatively affects the postharvest quality of apples.

Controlling ethylene production is a key method to extend the storage life of apples. Various approaches can limit ethylene production and slow the ripening process. This can be achieved by using compounds like 1-methylcyclopropene (1-MCP) or by reducing the respiration rate through a low-O_2_ and/or high-CO_2_ atmosphere. Mantaining a low-O_2_ partial pressure (<2.0 kPa) and a high-CO_2_ partial pressure (≥1.0 kPa) decreases ethylene production and respiration, preserving the physicochemical characteristics of apple and inhibiting or reducing the occurrence of certain physiological disorders [21].

The aim of this study was to evaluate and compare the effects of 1-MCP postharvest treatment, controlled atmosphere and normal atmosphere storage on the quality attributes of ‘Golden Delicious’ and ‘Red Delicious’ apples. The work examined the impact of 5 months of storage on essential parameters such as dry weight, total soluble solids and titratable acidity, as well on colour, firmness, malic acid content, sugar composition and content and volatilome to assess changes in fruit composition and quality. Given that VOCs are produced and emitted through cellular metabolism, the volatilomic profile can be utilized to establish a distinctive volatilomic biosignature for these apples under different storage conditions.

## 2. Results

### 2.1. Effect of Storage Conditions on Firmness, Colour, Total Soluble Solids, Titratable Acidity and pH

Table 1 shows the firmness, total soluble solids (TSS), titratable acidity (TA), pH and colour values of the ‘Golden Delicious’ (GD) and ‘Red Delicious’ (RD) varieties at harvest and after storage under the different storage conditions studied. No significant differences in fruit firmness were found in normal atmosphere (NA) and controlled atmosphere (CA) storage compared to harvest for both apple varieties. On the other hand, there was a 28% increase in firmness in ‘Golden Delicious’ and about 22% in ‘Red Delicious’ when apples were treated with 1-MCP. The total soluble solids (TSS) values under normal atmosphere storage for both varieties did not undergo significant changes, while under controlled atmosphere storage, only the ‘Red Delicious’ variety showed a significant increase in TSS value (Table 1). The TSS values of ‘Golden Delicious’ and ‘Red Delicious’ did not show significant differences when apples were treated with 1-MCP.

Titratable acidity (TA) values decreased significantly in both varieties under normal atmosphere and controlled atmosphere storage compared to harvest (Table 1). In controlled atmosphere storage, the decrease in TA was greater, with losses between 33% and 66% for ‘Red Delicious’ and ‘Golden Delicious’, respectively. With the application of 1-MCP, a significant decrease of 28% and 22% in TA was observed in ‘Golden Delicious’ and ‘Red Delicious’ varieties, respectively. It was observed that under these conditions, there was a more effective delay in the loss of TA than under normal atmosphere and controlled atmosphere storage conditions.

The pH values increased significantly or remained constant in both varieties of apples under normal atmosphere and controlled atmosphere storage conditions (Table 1). The pH values for apples treated with 1-MCP behaved differently in the ‘Golden Delicious’ and ‘Red Delicious’ varieties. When compared to the pH values at harvest, the values in the ‘Golden Delicious’ variety had a significant increase of 9%, behaving in the same way as in normal atmosphere and controlled atmosphere storage. For the ‘Red Delicious’ variety, there were no significant differences in the pH for all storage conditions.

The luminosity values, when compared to the harvest (H) for the ‘Golden Delicious’ (73.9) and ‘Red Delicious’ (35.7) varieties, did not undergo significant changes when stored in a controlled atmosphere. When treated with 1-MCP, the apples’ luminosity values showed significant differences in the ‘Golden Delicious’ (71.2) and ‘Red Delicious’ (38.4) varieties. The chroma values of the fruits stored in a controlled atmosphere in the ‘Golden Delicious’ variety (54.2) increased by 14%, while in the ‘Red Delicious’ variety (34.2), they decreased by 13%. The differences were significant in both varieties. For the apples treated with 1-MCP, the variation was not significant in the ‘Golden Delicious’ variety, while in the ‘Red Delicious’ variety, there was a significant decrease of 11% in the chroma value. As with the luminosity values, the chroma values for apples treated with 1-MCP behaved differently in the two varieties. There were no significant differences in the hue value of the fruits stored in a controlled atmosphere and treated with 1-MCP in the ‘Golden Delicious’ and ‘Red Delicious’ varieties.

### 2.2. Effect of Storage Conditions on the Malic Acid and Sugars Content

The malic acid content and composition of soluble sugars (sorbitol, glucose, sucrose and fructose) in ‘Golden Delicious’ and ‘Red Delicious’ varieties under different storage conditions are presented in Table 2. There were no significant differences in the malic acid content of apples under normal atmosphere storage conditions for either ‘Golden Delicious’ or ‘Red Delicious’ compared to harvest values. A decrease in the malic acid content was observed in the ‘Golden Delicious’ and ‘Red Delicious’ varieties, by 12% and 18% respectively, under controlled atmosphere storage conditions, with a significant difference noted in the ‘Golden Delicious’ variety. When treated with 1-MCP, both ‘Golden Delicious’ and ‘Red Delicious’ varieties exhibited the lowest malic acid content compared to other storage conditions, decreasing by 31% and 30% respectively.

In ‘Golden Delicious’ and ‘Red Delicious’ varieties, there is a decrease in the total soluble sugars when stored in a normal atmosphere (32% and 26%, respectively), related to the decrease in fructose (34% and 33%, respectively), glucose (26% and 32%, respectively) and also sucrose (34% and 5%, respectively) (Table 2). In controlled atmosphere storage, there was a significant decrease in sucrose content compared to harvest, and on average for ‘Golden Delicious’, it was significantly lower than that found in apples stored under normal atmosphere for ‘Red Delicious’. Nevertheless, for ‘Red Delicious’, the decrease in sucrose was accompanied by a significant increase in both fructose and glucose, resulting in only a slight decrease in the total sugars. When apples were treated with 1-MCP for the ‘Golden Delicious’ variety, the total soluble sugar content remained unchanged, although a decrease in sucrose (53% decrease) and an increase in glucose (58% increase) content were observed compared to harvest.

For the ‘Red Delicious’ apple variety, the same trend for glucose (22% increase) and sucrose (80% decrease) as that described for ‘Golden Delicious’ was observed; nevertheless, there was a significant decrease in the total soluble sugar content (21% decrease) related to the significant decrease in the fructose content (14% decrease). No significant changes in the content of sorbitol were observed for both apple varieties under the three storage conditions.

### 2.3. Effect of Storage Conditions on the Volatilome of Whole Apples

Table 3 and Figures S1 and S2 depict the VOCs detected in the volatilome of ‘Golden Delicious’ and ‘Red Delicious’ apple varieties at harvest and after storage in normal atmosphere, controlled atmosphere and apples treated with 1-MCP before storage in a controlled atmosphere. A total of 31 VOCs were identified in the volatilome of the two apple varieties under study in the different storage conditions, including 28 esters, one phenol and two terpenoid compounds. The volatilome obtained for ‘Golden Delicious’ [22,23,24,25] and ‘Red Delicious’ [26,27] whole apples is in accordance with previous works.

At harvest, esters were the most dominant group of VOCs, accounting for 57% and 52% of the total area for ‘Golden Delicious’ and ‘Red Delicious’ apple varieties, respectively. Hexyl 2-methylbutanoate (29% and 36% of total ester abundance), hexyl hexanoate (14% and 13% of total ester abundance), butyl hexanoate (14% and 11% of total ester abundance), hexyl acetate (14% and 6% of total ester abundance) and hexyl butanoate (10% and 4% of total ester abundance) were the most abundant esters in ‘Golden Delicious’ and ‘Red Delicious’ apples at harvest, representing 81% and 70% of the total ester abundance, respectively. For ‘Red Delicious’ apples, other esters were detected compared to ‘Golden Delicious’, including ethyl butanoate, propyl propionate, ethyl 2-methylbutanoate, propyl 2-methylbutanoate, propyl hexanoate, pentyl pentanoate, ethyl octanoate, pentyl hexanoate and propyl octanoate, which represent 10% of the total esters determined for the ‘Red Delicious’ apple variety. At harvest, (*E*,*E*)-α-farnesene was found to be the most abundant VOC in both apple varieties, representing 43% and 48% of the total area for ‘Golden Delicious’ and ‘Red Delicious’, respectively. Although a decrease in abundance was also observed during storage, apples treated with 1-MCP presented the lowest abundance of (*E*,*E*)-α-farnesene for ‘Golden Delicious’ (83% decrease compared to harvest). The decrease observed for normal and controlled atmosphere was lower (69% decrease). For ‘Red Delicious’, normal atmosphere storage resulted in the lowest abundance of (*E*,*E*)-α-farnesene (76% decrease). On the other hand, the treatment of apples with 1-MCP resulted in only a 38% decrease in (*E*,*E*)-α-farnesene, and under controlled atmosphere conditions, the decrease was also lower than that observed for ‘Golden Delicious’ (56% decrease). The ‘Red Delicious’ variety, which is highly susceptible to developing superficial scald, showed a higher chromatographic area of (*E*,*E*)-α-farnesene than the ‘Golden Delicious’ variety with the application of 1-MCP, and this was significantly different from the storage conditions in normal atmosphere and controlled atmosphere.

**Figure 1:**
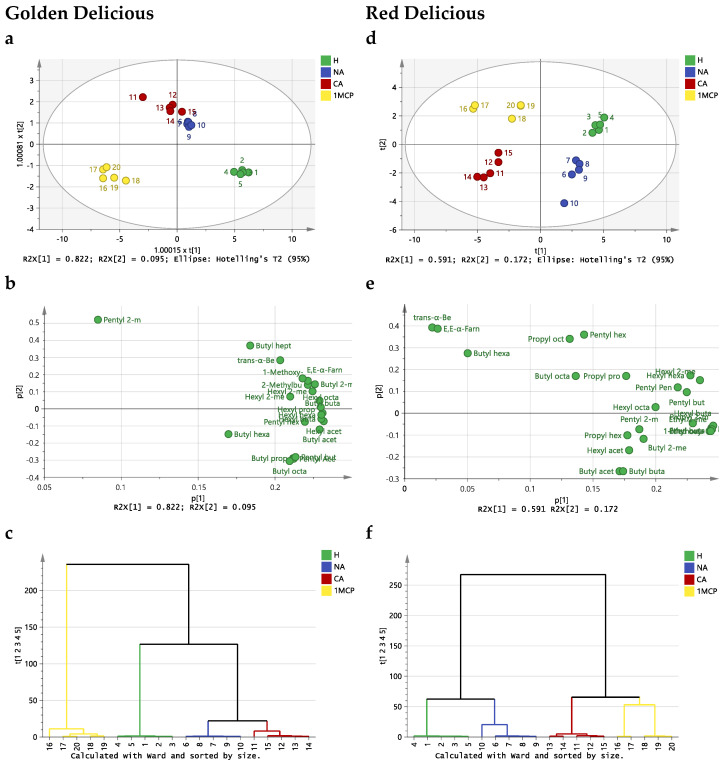
Principal component analysis score scatter plot (**a**,**d**) and loadings (**b**,**e**) originating from the volatilome of ‘Golden Delicious’ (**a**,**b**) and ‘Red Delicious’ (**d**,**e**) apples at harvest (H) and stored under normal atmosphere (NA), controlled atmosphere (CA) and controlled atmosphere after treatment with 1-MCP (1-MCP). Hierarchical cluster analysis using the Ward distance of the PC1 and PC2 score plots for ‘Golden Delicious’ (**c**) and ‘Red Delicious’ (**f**) apples.

A PCA model was initially constructed based on the volatilome for each apple cultivar to enable the identification of clusters, groups or outliers and ascertain the differences between the three storage conditions and also the change in apple volatilome in relation to the harvest date (Figure 1). For ‘Golden Delicious’ apples (Figure 1a), the first two PCs accounted for a total variance of 91.7% (the first principal component, PC1, accounted for 82.2%, while the second principal component, PC2 contributed to 9.5%). The overview of the two-dimensional score plot (Figure 1a) shows the clear formation of four groups, which implies that there were indications of grouping among different storage conditions. This grouping was supported by the cluster analysis of the sample scores on the plane defined by PC1 × PC2 (Figure 1b). Samples were arranged in PC1 with decreasing abundance of all the apple volatilome (Figure 1a,c).

For ‘Red Delicious’ apples, the first two PCs accounted for a total variance of 76.3% (PC1 accounted for 59.1% of the total variance, while PC2 contributed to 17.2%). The overview of the two-dimensional score plot (Figure 1d) also shows, for ‘Red Delicious’, the clear formation of four groups, which implies that there were indications of grouping among different storage conditions.

**Figure 2:**
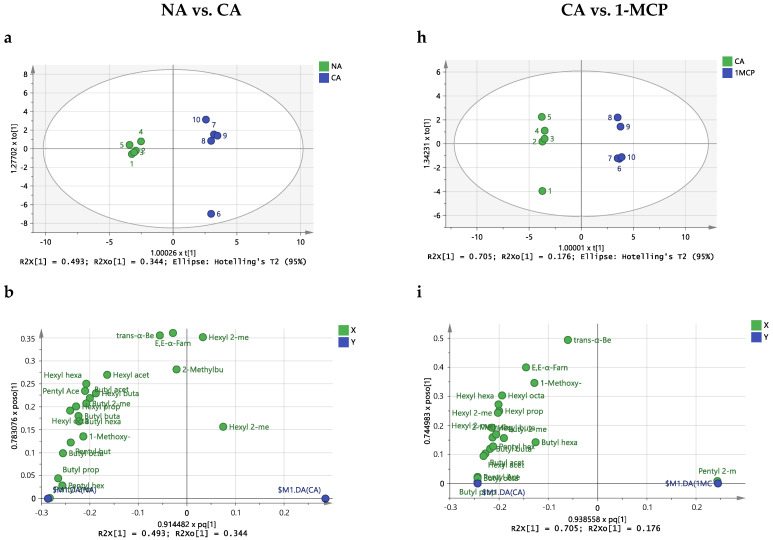

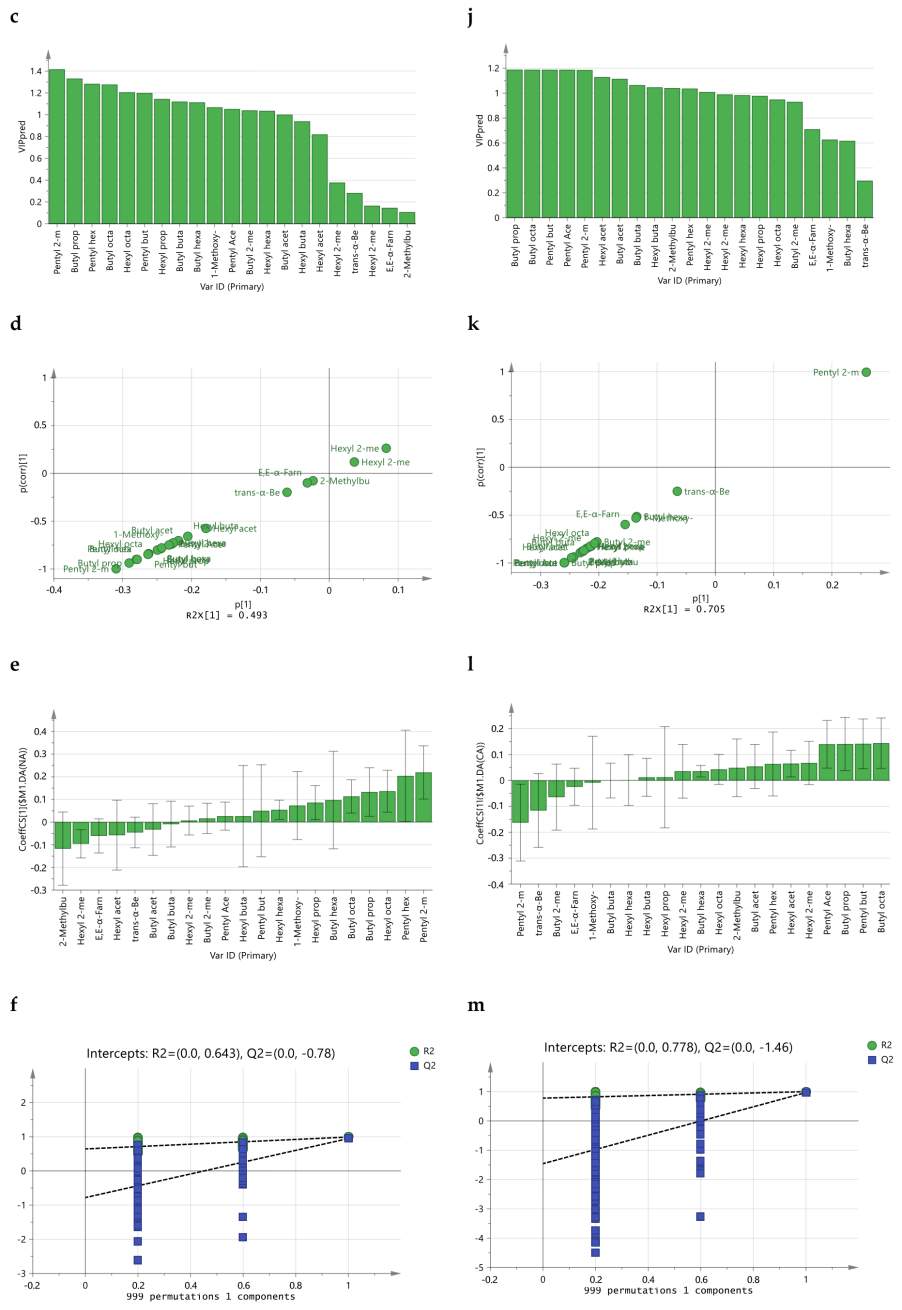

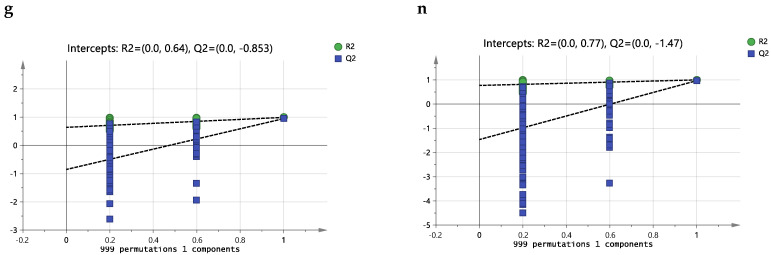
The OPLS-DA analysis of the volatilome of ‘Golden Delicious’ under different storage conditions. (**a**) score plot for NA vs. CA; (**b**) loading plot between the X-variables and the Y-variables for the first predictive component and the first Y-orthogonal component for NA vs. CA (**c**) The VIP (variable importance for the projection) for NA vs. CA; (**d**) S-Plot for NA vs. CA; (**e**) Beta-coefficients for NA vs. CA; (**f**,**g**) validation plot of 999 permutation tests for NA vs. CA. The slope of R^2^ > 0 together with the intercept of Q^2^ on the y-axis < 0 indicating a valid model. (**h**) score plot for CA vs. 1-MCP; (**i**) loading plot between the X-variables and the Y-variables for the first predictive component and the first Y-orthogonal component for CA vs. 1-MCP (**j**) The VIP (variable importance for the projection) for CA vs. 1-MCP; (**k**) S-Plot for CA vs. 1-MCP; (**l**) Beta-coefficients for CA vs. 1-MCP; (**m**,**n**) validation plot of 999 permutation tests for CA vs. 1-MCP. The slope of R^2^ > 0 together with the intercept of Q^2^ on the y-axis < 0 indicating a valid model.

This grouping was supported by the cluster analysis of the sample scores on the plane defined by PC1 × PC2 (Figure 2e). Samples were arranged in PC1 with decreasing abundance of all the apple volatilome (Figure 2d,f). The results indicated that for both apples, the volatilome at harvest was clearly separated from the other storage conditions on PC1, with the apples treated with 1-MCP and controlled atmosphere storage being clearly separated from apples stored in normal atmosphere. The volatilome of ‘Golden Delicious’ and ‘Red Delicious’ apples at harvest was the most abundant in all VOCs, with the volatilome of the apples treated with 1-MCP and controlled atmosphere storage presenting the lowest abundance of all detected VOCs.

To uncover the underlying differences between storage conditions for ‘Golden Delicious’ and ‘Red Delicious’ apples, an OPLS-DA was applied between the volatilome of apples after storage in normal atmosphere and controlled atmosphere and between the volatilome of apples stored after controlled atmosphere vs. 1MCP. This would help disclose any potential critical VOCs among the three storage conditions.

**Figure 3:**
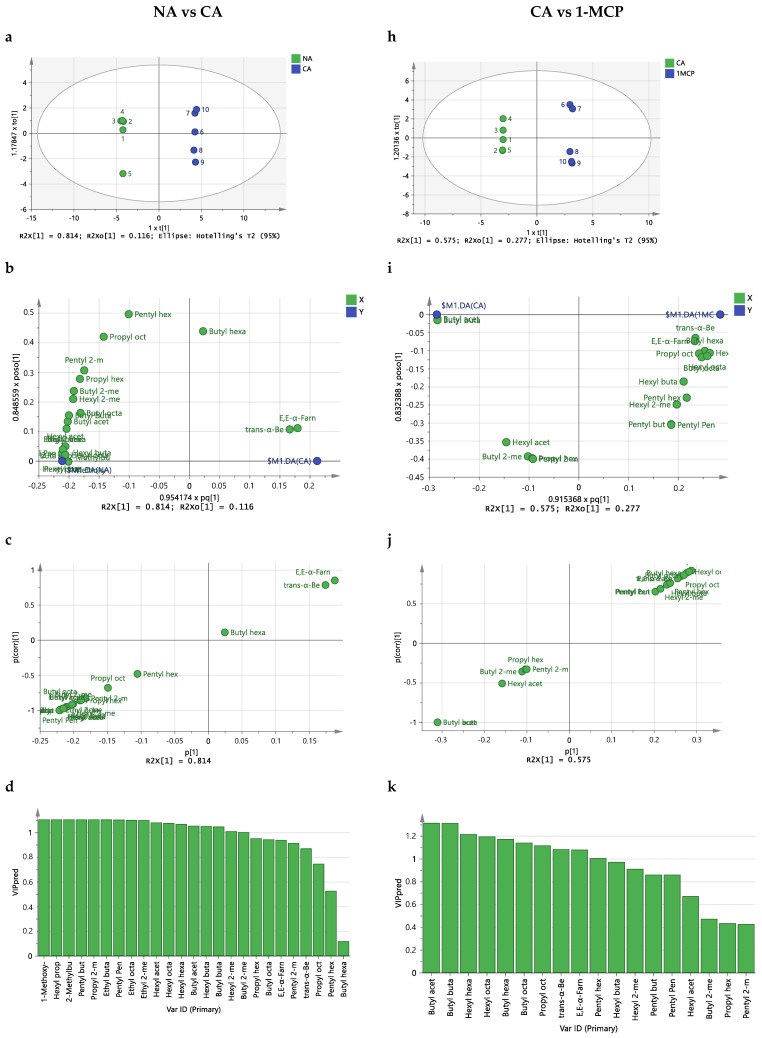

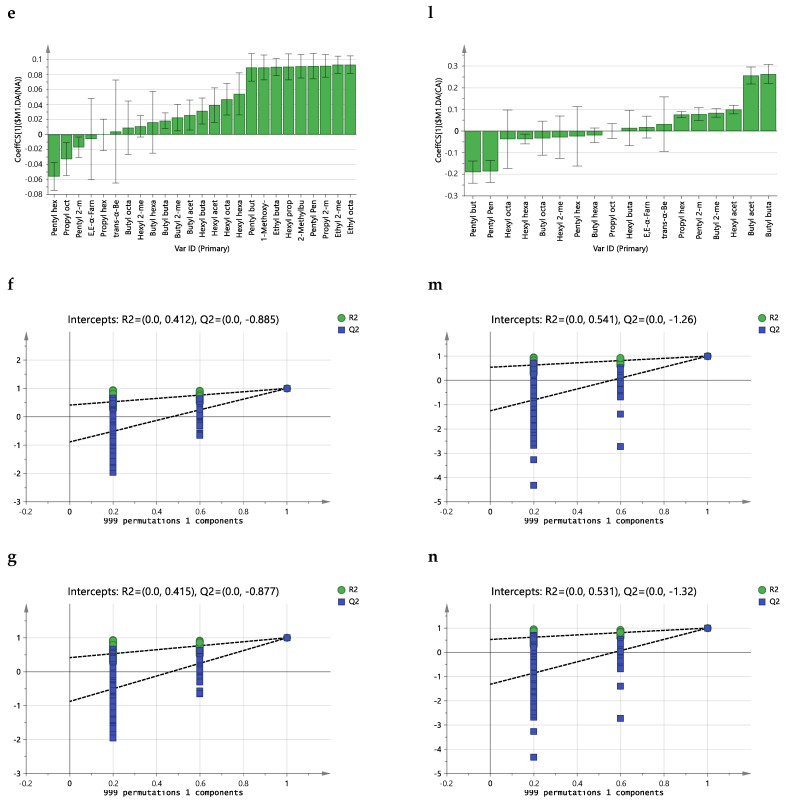
The OPLS-DA analysis of the volatilome of ‘Red Delicious’ under different storage conditions. (**a**) Score plot for NA vs. CA; (**b**) loading plot between the X-variables and the Y-variables for the first predictive component and the first Y-orthogonal component for NA vs. CA (**c**) The VIP (variable importance for the projection) for NA vs. CA; (**d**) S-Plot for NA vs. CA; (**e**) Beta-coefficients for NA vs. CA; (**f**,**g**) validation plot of 999 permutation tests for NA vs. CA. The slope of R^2^ > 0 together with the intercept of Q^2^ on the y-axis < 0 indicating a valid model. (**h**) Score plot for CA vs. 1-MCP; (**i**) loading plot between the X-variables and the Y-variables for the first predictive component and the first Y-orthogonal component for CA vs. 1-MCP. (**j**) The VIP (variable importance for the projection) for CA vs. 1-MCP; (**k**) S-Plot for CA vs. 1-MCP; (**l**) Beta-coefficients for CA vs. 1-MCP; (**m**,**n**) validation plot of 999 permutation tests for CA vs. 1-MCP. The slope of R^2^ > 0 together with the intercept of Q^2^ on the y-axis < 0 indicating a valid model.

For ‘Golden Delicious’ apples, the two models (Figure 2) were established with one predictive and two and three orthogonal components for the normal atmosphere vs controlled atmosphere and controlled atmosphere vs 1-MCP comparisons. The R^2^Y and Q^2^Y for the two models were all satisfactory, with R^2^Y > 0.999 and Q^2^Y > 0.947. Permutation test results showed the quality and predictive ability of the models obtained (no overfitting), indicating that the models were suitable for discriminating and seeking potentially critical VOCs (Figure 3).

To reveal potential differential VOCs among the three storage conditions, the following results were taken into consideration. Firstly, the S-plot was employed for exploring potential critical VOCs [28]. In this study, the criteria for the S-plot were set with |p(cov)| ≥ 0.2 and |p(corr)| ≥ 0.7. Secondly, the variable importance (VIP), expressing the contribution of each variable, was utilized to seek potential aromas with a threshold greater than 1 [29]. Third, the regression coefficients significantly different from zero and fourth, the fold change (FC) and *p*-values (after Bonferroni correction).

As depicted in Figure 3b of the S-plot, fourteen compounds were identified as potential differential compounds responsible for the paired comparison of normal atmosphere and controlled atmosphere. Regarding the VIP, also fourteen compounds were filtered as candidates to characterize the differences with VIP > 1, although they were not all the same. For the regression coefficients, only eight variables were considered significant in the separation between the two groups. Taking also into consideration the FC and t-test *p*-values, five VOCs—butyl acetate, 2-methylbutyl acetate, butyl propionate, pentyl acetate and hexyl butanoate—were considered as potential critical VOCs for differentiating the volatilome of ‘Golden Delicious’ apples stored under normal atmosphere and controlled atmosphere. Similarly, for the paired comparison of controlled atmosphere and 1-MCP, six VOCs were screened as potentially critical VOCs, namely pentyl 2-methylbutanoate, hexyl acetate, pentyl acetate, butyl propionate, pentyl butanoate and butyl octanoate, by means of the S-plot, VIP, regression coefficients, FC and *p*-value (Figure 3g–l).

For ‘Red Delicious’ apples, when comparing storage under normal atmosphere with controlled atmosphere using the same methodology, sixteen VOCs were found to differentiate these two storage conditions. These VOCs include 1-Methoxy-4-(2-propen-1-yl)-benzene (Estragol), hexyl propionate, 2-methylbutyl acetate, pentyl butanoate, propyl 2-methylbutanoate, ethyl butanoate, pentyl pentanoate, ethyl octanoate, ethyl 2-methylbutanoate, hexyl acetate, hexyl octanoate, hexyl hexanoate, butyl acetate, hexyl butanoate, butyl butanoate and butyl 2-methylbutanoate. For the comparison of ‘Red Delicious’ storage in controlled atmosphere vs 1-MCP, only two VOCs were found to be critical: butyl butanoate and butyl acetate.

## 3. Discussion

Storage of apples under normal atmosphere and controlled atmosphere allowed for the retention of firmness in both ‘Golden Delicious’ and ‘Red Delicious’ apples stored for 5 months. Additionally, the use of 1-MCP before controlled atmosphere storage resulted in an increase in apple firmness for both apple cultivars. This increase enhances desirable characteristics in the fruit for the market, improving its texture quality and consumer acceptance [3,30]. The low temperature, high relative humidity and high-CO_2_ and low-O_2_ partial pressure used in CA reduce ethylene biosynthesis [31]. On the other hand, 1-MCP can block ethylene binding sites [32], inhibiting and delaying ethylene-dependent responses, including respiration, ripening rate, internal ethylene concentration and loss of firmness. Loss of apple firmness during storage has been shown to be related to cell wall pectin depolymerization [33,34,35,36]. It has been demonstrated that the use of 1-MCP reduces the activity of four important enzymes involved in pectin modification (exo-polygalacturonase, pectin methylesterase, β-galactosidase and α-L-arabinofuranosidase). Furthermore, 1-MCP treatment reduces the expression of pectin-degrading candidate genes (MdPG1, MdPME1, Mdβ-GAL1, Mdβ-GAL2 and Mdα-ARF2) [37]. In relation to apple firmness, both apple cultivars exhibited the same behavior under the different storage conditions studied.

Storage of ‘Golden Delicious’ and ‘Red Delicious’ apples under normal atmosphere resulted in a significant decrease in the levels of glucose, fructose and sucrose and consequently in the total soluble sugars. On the other hand, storage of ‘Golden Delicious’ and ‘Red Delicious’ under controlled atmosphere resulted in an increase in glucose, a decrease in sucrose and fructose, resulting in a small but significant decrease in total soluble sugars. 1-MCP treatment before controlled atmosphere storage resulted in a more significant decrease in sucrose levels compared to controlled atmosphere, an increase in glucose and a decrease in fructose for ‘Red Delicious’ and an increase of fructose for ‘Golden Delicious’. The reduced loss of fructose and glucose in controlled atmosphere and 1-MCP-treated ‘Golden Delicious’ and ‘Red Delicious’ apples can be attributed to suppressed respiration, which is a consequence of controlled atmosphere and 1-MCP storage conditions.

The effect of storage conditions, normal atmosphere and controlled atmosphere, as well as the use of 1-MCP on the total soluble sugars of apples after storage, has been inconsistent. Suni et al. [38] observed a small decrease or no change in the total soluble sugar content for ‘Summerred’, ‘Belle de Boskoop’ and ‘Mutzu’ and a decrease for ‘Belle de Boskoop’ and ‘Jonagold’ apple varieties stored in refrigerated normal atmosphere for approximately 4 months. However, in all varieties, they observed a decrease in sucrose content that was compensated by an increase in fructose content. For glucose, they observed that it did not change or decreased slightly. Roth et al. [39] observed a decrease in sucrose during storage in normal atmosphere (1 °C, 21 kPa O_2_, 0.03 kPa CO_2_) and under controlled atmosphere (1 °C, 1 kPa O_2_, 2.5 kPa CO_2_) for 6 months in ‘Jonagold’ apple variety. At the same time, glucose and sorbitol contents showed an increase as a function of storage time. Zhu et al. [40] also observed a higher decrease in sucrose in normal atmosphere storage (0 °C, 95% relative humidity) compared to controlled atmosphere storage (2 kPa O_2_ + 1 kPa CO_2_ at 0 °C) during 4 months and 8 months of storage for ‘Fuji’ samples. They also observed a significant increase in glucose and fructose in controlled atmosphere storage compared to normal atmosphere storage and harvest. Expression levels of key genes, such as sucrose synthase (SS), sucrose phosphate synthase (SPS), fructokinase (FK) and hexokinase (HK), were shown to be correlated with the corresponding enzyme activities. They found that activities of neutral invertase (NI), vacuolar invertase (VI), FK and HK were inhibited, but SPS activity was promoted in apple fruit stored in controlled atmosphere, suggesting that controlled atmosphere storage could enhance sucrose synthesis and delay hydrolysis of sucrose and hexose. 1-MCP has been described as having little or no effect on the total soluble sugars [41,42,43,44], increasing soluble sugars after treatment with 1-MCP [45], or resulting in lower levels of total soluble sugars [46,47].

Sucrose, with a sweetness value of 100, is considered the standard for comparing the sweetness levels of different sugars [48]. Accordingly, the respective relative sweetness values of fructose, glucose and sorbitol are 150–170, 70–80 and 55–70 [49]. As the sweetness levels of individual sugars differ, the sweetness index for ‘Golden Delicious’ and ‘Red Delicious’ apples after normal atmosphere storage shows an average decrease of 32%. The decrease was smaller for apples stored in controlled atmosphere (an average of 11%) and previous treatment with 1-MCP followed by storage in controlled atmosphere did not change the sweetness index for ‘Golden Delicious’ apples, but for ‘Red Delicious’ apples, there was a decrease of 20%. On the other hand, for apple storage under a normal atmosphere, there was a decrease of nearly 30% in total sugars, which can have a positive nutritional effect. In fact, for both apple varieties stored in normal atmosphere, there was a decrease in the glycemic load (GL) of apples by 32% for ‘Golden Delicious’ and 25% for ‘Red Delicious’ (Table 2). The concept of glycemic load (GL) was introduced to account for the contribution not only of the type of carbohydrate (i.e., glycemic index) but also the amount of available carbohydrates per serving to the overall glycemic response [50].

Using 1-MCP before storage under controlled atmosphere resulted in higher TA when compared to controlled atmosphere storage alone for ‘Golden Delicious’ apples but not for ‘Red Delicious’ apples, although all storage conditions resulted in significantly lower TA for both apple cultivars when compared to harvest. A number of studies have demonstrated the effectiveness of 1-MCP in suppressing or delaying the loss of total acidity (measured as TA) during the storage of apples. However, the extent of TA loss suppression depends on time, storage conditions and other related postharvest factors [22,42,44,45,51,52,53,54,55]. It is expected that this decrease in total acidity of apples during storage will result in a change in the acid taste sensations of apples, as a positive correlation has been described between TA and tart, sour, or acid taste sensations [56]. According to Harker et al. [57], TA differences as low as 0.08 g/100 mL evoke a response in perceived acid taste. Therefore, the change in the perceived acid taste sensation is expected to be higher in ‘Golden Delicious’ apples (a change of 2.2 g/100 mL for 1-MCP treated apples versus harvest) than in ‘Red Delicious’ apples (a change of 1.0 g/100 mL for 1-MCP treated apples versus harvest).

For 1-MCP treated apples only, a significant decrease in malic acid content was observed in both ‘Golden Delicious’ and ‘Red Delicious’ apples. For normal atmosphere and controlled atmosphere storage, there were no significant reductions in malic acid content for both apple cultivars, although on average, controlled atmosphere storage resulted in a decrease in malic acid. Nevertheless, the decrease in malic acid during normal atmosphere storage and controlled atmosphere storage has been described for several apple cultivars [38,39,58]. On the other hand, Liu et al. [59] found that 1-MCP maintained the malate content of ‘Fuji’ apples stored in air at 20 °C for 56 days, and Lee et al. [47] found the same trend for ‘Fuji’ apples stored at 2 °C for 9 months in normal atmosphere. Nevertheless, it is interesting to note that there was a significant inverse correlation between the malic acid content of apples and their glucose levels (r = 0.933, *p* < 0.000715, n = 8) and a positive correlation between malic acid and sucrose content in ‘Red Delicious’ (r = 0.983, *p* < 0.018, n = 4). In one study comprising 364 apple accessions, malic acid content was positively correlated with glucose content and negatively correlated with sucrose content [60]. In another study comprising the fruit of 155 apple accessions, the malic acid content showed a negative correlation with glucose content, whereas glucose content negatively correlated with sucrose content [61]. The potential crosstalk between organic acid and sugar metabolism in fruit remains rather limited [62]. The decrease in malate content during fruit ripening has usually been attributed to its degradation by cytosolic NADP-dependent malic enzyme (cyNADP-ME, EC 1.1.1.40) [63,64]. In addition, malate serves as a substrate for the gluconeogenesis pathway to produce glucose, by converting malic acid to phosphoenolpyruvate via oxaloacetic acid through the action of phosphoenolpyruvate carboxylase kinase (PEPCK, EC 4.1.1.49) [65].

For ‘Golden Delicious’ and ‘Red Delicious’ apples, there was a significant reduction in ester VOCs in all storage conditions compared to harvest, in accordance with literature [31,66,67,68,69]. This is explained by the reduced levels of ethylene in normal atmosphere and controlled atmosphere storage, as well as the inhibitory effect of 1-MCP [70]. In a controlled atmosphere compared to a normal atmosphere, there was a significant decrease in ester VOCs for both cultivars, although the reduction was more pronounced for ‘Red Delicious’ apples. It is known that low pO_2_ reduces ethylene biosynthesis and consequently the activity of enzymes that produce VOCs, such as lipoxygenase (LOX), alcohol dehydrogenase (ADH) and alcohol acyltransferase (AAT). Several authors observed this reduction of VOCs during controlled atmosphere storage compared to cold storage [31,66,67,68,71,72]. Nevertheless, reduction in ester VOCs can also be explained by a limited availability of precursors such as free fatty acids [67,73,74,75]. Furthermore, it has been shown that 1-MCP and ethylene generally produce opposite effects on genes related to apple volatile biosynthesis [76]. Application of 1-MCP in ‘Golden Delicious’ and storage under normal atmosphere at 20 °C for 43 days had a significant effect on the expression of amino acid catabolism genes, BCATs (branched-chain amino acid aminotransferase), ArAT (aromatic amino acid aminotransferase) and AADC (amino acid decarboxylases), three genes that may be involved in the initial conversion of amino acids to aroma compounds, significantly up-regulating the expression of MdBCAT2 gene and reducing the expression of MdArAT and MdAADC. A reduced expression of genes involved in fatty acid biosynthesis and the lipoxygenase (LOX) pathway, MdACPs (acyl-carrier-proteins), MdMCAT (Malonyl-CoA:ACP transacylase) and MdACDP (Acyl-ACP-desaturase), was also observed after treatment with 1-MCP. 1-MCP treatment markedly suppressed the expression of MdLOX, a gene involved in volatile biosynthesis derived from fatty acids. Expression of both MdAAT1 and MdAAT2 (alcohol acyltransferases) was strongly reduced in 1-MCP treated fruit. Overexpression of the MdAAT2 gene alters the profile of volatiles, while depression of MdAAT2 prevented subsequent ester production [73]. AAT is able to use a wide range of alcohol substrates and acyl donors to make many esters [77], although the final volatile profile is dependent on precursor formation and availability [9].

However, no significant effect of treatment with 1-MCP on the expression of MdACAS, MdECH, MdACAD or MdKAT genes involved in the fatty acid β-oxidation pathway was observed [16]. Nevertheless, when compared to storage under controlled atmosphere, ‘Golden Delicious’ and ‘Red Delicious’ responded differently to the application of 1-MCP in the abundance of ester VOCs emitted by whole apples. While for ‘Golden Delicious’, the application of 1-MCP and storage under controlled atmosphere resulted in the lowest abundance of ester VOCs of all storage conditions studied, for ‘Red Delicious’, the use of 1-MCP and storage under controlled atmosphere resulted in a significantly higher level of ester VOCs compared to storage under controlled atmosphere without the use of 1-MCP. This increase in ester VOCs in ‘Red Delicious’ apples was related to the increased abundance of hexanoate esters compared to controlled atmosphere. The different behavior of hexanoate esters production in ‘Red Delicious’ apples observed for 1-MCP treated apples, contrary to that observed for the other esters, may be due to a different substrate specificity of the ‘Red Delicious’ AAT enzyme [15] or substrate availability [67,73,74,75]. Additionally, it has been shown that the differential expression of AAT genes can contribute to the phenotypic variation of volatile ester biosynthesis, and postharvest 1-MCP treatment showed selected inhibition on gene expression of specific AAT and ACS family members [25].

Several studies have demonstrated that the level of internal ethylene concentration (IEC) in fruit at the time of 1-MCP treatment is a crucial factor for the effectiveness of 1-MCP in apples [77,78,79,80]. The higher IEC, which is commonly observed in late-harvested apples [78], could compete for the binding sites on ethylene receptors, making the fruits less responsive to 1-MCP application. Even if 1-MCP binds permanently to receptors present at the time of treatment, the possibility of regenerating new receptors has been suggested [41].

2-Methylbutyl acetate, butyl acetate and hexyl acetate are high-impact esters regarding apple flavour [23,25,81,82]. For these esters, 1-MCP had the same effect on both apple varieties compared to storage under controlled atmosphere. Also, for both apples stored under normal atmosphere, controlled atmosphere and 1-MCP resulted in lower levels of 2-methylbutyl acetate, butyl acetate and hexyl acetate. In fact, for ‘Golden Delicious’ and ‘Red Delicious’ apples, when comparing storage under normal atmosphere vs. controlled atmosphere, 2-methylbutyl acetate and butyl acetate were critical VOCs for differentiating these two storage conditions, and for ‘Golden Delicious’, hexyl acetate was also found as a critical VOC when storage under controlled atmosphere vs. 1-MCP is considered and butyl acetate for the comparison between controlled atmosphere vs. 1-MCP for ‘Red Delicious’, although in this case, 1-MCP treatment resulted in higher levels of this VOC.

It has been described that generally, branched-chain esters’ concentration is not affected by storage in low or extremely low pO_2_ [69,83]. Contrary to what has been shown for ‘Braeburn’ [84], ‘Royal Gala’ [69] and ‘Mondial Gala’ [83] apples, the concentration of 2-methylbutyl acetate was affected by storage conditions or 1-MCP application for both ‘Golden Delicious’ and ‘Red Delicious’ apple cultivars.

Although the majority of aroma compounds from apples are volatile esters, apples also produce a relatively large amount of (*E*,*E*)-α-farnesene. For the abundance of (*E*,*E*)-α-farnesene, again, there was observed a different behavior between ‘Golden Delicious’ and ‘Red Delicious’ after 1-MCP application and also for storage under normal atmosphere and controlled atmosphere. For ‘Golden Delicious’ apples, all storage conditions resulted in a decrease in (*E*,*E*)-α-farnesene, with the use of 1-MCP resulting in a lower abundance of (*E*,*E*)-α-farnesene. Nevertheless, for ‘Red Delicious’, normal atmosphere storage resulted in the lowest abundance of (*E*,*E*)-α-farnesene, and ‘Red Delicious’ apples treated with 1-MCP resulted only in a 30% decrease in the abundance of (*E*,*E*)-α-farnesene, contrasting with the 87% decrease observed for ‘Golden Delicious’ apples treated with 1-MCP.

The impact of storage conditions on the abundance of (*E*,*E*)-α-farnesene is important, as although (*E*,*E*)-α-farnesene with its green, oily and fatty notes contributes mainly to the vegetable descriptor of apple fruit [85], the products of (*E*,*E*)-α-farnesene oxidation could induce scald disorder [86,87,88]. The superficial scald of fruit is induced with prolonged storage at low temperature, and its severity is related to the storage temperature [89]. Scald susceptibility of apple cultivars is a function of (*E*,*E*)-α-farnesene production and its oxidation to conjugated trienols [90]. Studies have demonstrated that the use of 1-MCP in apple storage leads to a decrease in the compound (*E*,*E*)-α-farnesene. Watkins et al. [51] found a reduction in the ‘Law Rome’ variety, and Shaham et al. [91] observed a decrease in the ‘Granny Smith’ variety. Inhibition of superficial scald using 1-MCP has been linked to the inhibition of (*E*,*E*)-α-farnesene accumulation, restricting the substrate for oxidation, as shown by several studies [22,42,51,92]. Therefore, while the use of 1-MCP was beneficial for lowering the ‘Golden Delicious’ apple (*E*,*E*)-α-farnesene abundance, its action on ‘Red Delicious’ apples was more limited. The higher levels of (*E*,*E*)-α-farnesene at harvest and in all storage conditions studied are consistent with the fact that ‘Red Delicious’ is one of the apple cultivars known to be more sensitive to developing this disorder [93]. Also, the success of 1-MCP for reducing superficial scald disorder is dependent on the cultivar [22,42,51,79,94,95]. In all the above studies, the effectiveness of 1-MCP treatment appears to be related to whether or not the inhibition of ethylene production is maintained during long-term storage. Hence, repetition of 1-MCP treatments in long-term storage of ‘Red Delicious’ seems to be recommended for the continuity of 1-MCP activity [41].

## 4. Materials and Methods

### 4.1. Chemicals

Both the SPME holder for manual sampling and the 100 μm polydimethylsiloxane (PDMS) fibre used in the analyses for were purchased from Supelco (Aldrich, Bellefonte, PA, USA). The standards of ethyl butanoate (>98.0%), ethyl 2-methylbutanoate (>98.0%), butyl acetate (>99.0%), butyl acetate (≥98.0%), pentyl acetate (≥98.5%), butyl butanoate (>99.0%), butyl 2-methylbutanoate (≥98.5%), hexyl acetate (>99.0%), pentyl butanoate (≥98.0%), ethyl octanoate (>99.0%) and (*E*,*E*)-a-Farnesene, sulphuric acid, glacial acetic acid, n-alkane series (C7–C30) were obtained from Sigma-Aldrich (Bellefonte, PA, USA). HPLC-grade water was prepared with the SGS water purification system. The regenerated cellulose membrane filters were from Marchery-Nigel (Düren, Germany).

### 4.2. Vegetable Material

In this study, two apple cultivars, ‘Golden Delicious’ and ‘Red Delicious’, grown in the region of Carrazeda de Ansiães, Portugal (41.2429° N, 7.3076° W), were used. The apples came from certified apple trees grafted on M9 rootstock in 7-year-old orchards, conducted on a vertical axis with a planting density of 4 × 1.2 m. The apples were harvested at commercial maturity, determined by visually rating the extent of starch hydrolysis on a 1–9 scale (1 = full starch, 9 = no starch) after staining an equatorial section of each apple with a 5 mg/L I–KI solution (n = 20). Apples were harvested with an average starch index of 4. For ‘Golden Delicious’, apples with a minimum firmness of 18 N were used, while for ‘Red Delicious’, those with a minimum firmness of 24 N were selected. The apples used in the study were harvested from 20 threes in the largest diameter of the apple tree, at chest height, and only apples from the exposed to the sun. The fruit were analysed at harvest, and samples of ‘Golden Delicious’ and ‘Red Delicious’ were kept for five months under three types of storage conditions: refrigerated atmosphere (RA) at 2 °C and 90% relative humidity, controlled atmosphere (CA) with pO_2_ 3 kPa and pCO_2_ 1.0 kPa, and postharvest treatment with 2.475 g of 1-methylcyclopropene (1-MCP) applied for 24 h in a volume of 2 m^3^, followed by storage in CA. After the 5-month storage period, samples were immediately analysed.

### 4.3. Firmness

The firmness of the apples was determined using a TA-XTPlus texture analyzer (Stable Micro Systems, Surrey, UK), with a 50 kg load cell. A cylindrical penetration probe with a diameter of 6 mm (P6) was used for the analysis at a speed of 2 mm/s with a displacement of 25 mm. Firmness was measured in the equatorial region of the whole fruits and the results were expressed in newtons (N).

### 4.4. Total Soluble Solids, pH, Total Acidity

Total soluble solids, expressed in %, were determined in apple juice using an Atago PR-101 refractometer (Atago, Co., Ltd., Tokyo, Japan) at 20 °C [96]. Titratable acidity was determined by titrating 10 mL of diluted juice in 100 mL of distilled water, with 0.1 N sodium hydroxide solution, to pH = 8.2. The results were expressed in g of malic acid/100 mL [97]. pH was determined in apple juice using a Jenway 3310 pH meter (Cole-Parmer Instrument Co., Ltd., Saint Neots, UK).

### 4.5. Chromatic Characteristics

The chromatic parameters were measured in four quadrants of the middle region of the fruit, using a Minolta CR-300 colourimeter (Konica Minolta, Inc., Tokyo, Japan). The CIELab colour space was used, and the colour was expressed in terms of luminosity (L*), chroma (C*) and hue angle (h°). Luminosity ranges from 0 (black) to 100 (white); chroma represents colour saturation; hue angle is expressed in degrees (°), with red being near 0°, yellow near 90°, green near 180°, and blue near 270° [96].

### 4.6. Determination of Malic Acid

Malic acid was extracted and quantified by adapting the method of Shui and Leong [98], with some modifications. Lyophilized apple pulp (0.5 g) was extracted in 5 mL of methanol/10% acetic acid (85/15) for 30 min in an ultrasound bath. The suspension was centrifuged for 5 min at 6000 rpm and the supernatant was recovered. For the analysis, an Aminex HPX-87H column (300 × 7.8 mm, 9 μm particle size, Bio-Rad, Hercules, CA, USA) was used. The optimal analysis conditions were achieved using a 5 mM sulfuric acid solution (B): ultrapure water (A) (20:80, *v*/*v*) for 30 min. The flow rate was 0.6 mL/min, and the absorbance was monitored between 210–350 nm, with the absorbance at 210 nm used for quantification. Quantification was performed by external calibration with a calibration curve in the 50 mg/L to 5000 mg/L range. Analyses were performed in quintuplicate.

### 4.7. Determination of Free Sugars by High-Performance Anion Exchange Chromatography with Pulsed Amperometric Detection

Samples were analysed using a method based on that of Vasconcelos et al. [99]. Freeze-dried samples (100 mg) were extracted with 9 mL 50% ethanol at 60 °C over 20 min. Afterwards, 1 mL internal standard (2-deoxyglucose, 2.5 mg/mL) was added followed by centrifugation (6000 rpm, 5 min, 20 °C). The supernatants were analysed by high-performance anion exchange chromatography (HPAEC, Dionex ICS 5000, Dionex, Sunnyvale, CA, USA) with pulsed amperometric detection (PAD) using a CarboPac PA-20 column (length 150 mm, width 3 mm, non-porous particles of 6.5 μm diameter covered with a thin layer of functionalized latex, Dionex, Sunnyvale, CA, USA) and a CarboPac PA-20 pre-column (30 × 3 mm, Dionex, Sunnyvale, CA, USA). An isocratic elution was used with a NaOH solution (10 mM) containing 2 mM Ba(OH)_2_ (to precipitate carbonate) at a flow rate of 0.35 mL/min. The eluent was kept under nitrogen atmosphere to reduce carbonate formation and biological contamination. The injection volume was 5 μL, and the column temperature was maintained at 35 °C during the separation. The electrochemical detector consisted of a gold (Au) working electrode, a silver/silver chloride (Ag/AgCl) reference electrode and a titanium counter electrode. The pulsed amperometric detection program was as follows: +0.1 V from 0.00 to 0.40 s, then −2.0 V from 0.41 to 0.42 s and a ramp from −2.0 to +0.6 V from 0.42 to 0.43 s, followed by −0.1 V from 0.44 to 0.50 s (end of the cycle). The integration was performed between 0.2 s to 0.4 s. The quantification was done by relating the recovery of the internal standard to a 100% control, recovery of added sugars in spiked samples and calibration curves for each of the analysed sugars (Fructose, Glucose, Sucrose and Sorbitol).

### 4.8. Estimation of Apple Taste Parameter

Total sugar content was indicated by the amount of three major sugars found in apple fruits, i.e., glucose, sucrose and fructose. Sweetness index for each apple accession was estimated according to a modified method as previously described by Ma et al. [60]. Briefly, the contribution of each major sugar found in apple fruits was calculated considering that fructose and glucose are 1.7 and 0.75 times sweeter than sucrose, respectively. As a result, sweetness index = 0.75 [Glucose] + 1.0 [Sucrose] + 1.7 [Fructose].

### 4.9. Glycemic Index (GI) and Glycemic Load (GL)

The predicted glycemic index (GI) of apples was calculated as recommended by the Food and Agriculture Organization/World Health Organization [100], by weighting the GI of each soluble carbohydrate in the apples:

GI = GIA × gA/g + GIB × gB/g + …

where GIA is the GI of component A, gA is the amount of available carbohydrate in component A (g), and g is the total amount of available carbohydrate in grams in the apples (50 g). The GI of single components was provided by Atkinson et al. [101]. High-GI foods are defined as those with a GI value ≥ 70, medium-GI foods are those with a GI from 56 to 69, and low-GI foods are those with a GI value ≤ 55 [102].

In order to calculate glycemic load (GL) values, a standardized available carbohydrate portion of 15 g carbohydrate was used and estimated the GL according to the formula [50],

GL = GI/100 × grams of carbohydrate in a standardized portion

### 4.10. Analysis of the Whole Apples Volatilome by HS-SPME/GC-MS

For headspace sampling, each apple weighing approximately 150 g was placed in a 1 L glass vial fitted with a Teflon lid. Consistency in the total weight and volume of the apples used in each analysis was maintained, as these parameters are crucial for the reproducibility of the methodology [103]. The extraction conditions followed those reported in the literature for the 100 μm polydimethylsiloxane (PDMS) fibre [104,105,106] and underwent a preliminary evaluation to ensure optimal analysis conditions in terms of chromatographic areas and signal-to-noise ratio. Among the evaluated fibres (PDMS, PDMS/DVB, CAR/PDMS and polyacrylate), PDMS exhibited the highest abundance of the target molecule, (*E*,*E*)-α-farnesene and demonstrated the best ester recovery, making it the ideal choice for this study.

To achieve equilibrium concentrations of volatiles in the headspace, the vials were left for 16 h. Subsequently, the vials were placed in a controlled temperature room set at 22.0 ± 1 °C for 24 h to ensure thermal equilibration and facilitate the transfer of compounds from the sample to the headspace. The SPME fibre, previously conditioned at 250 °C for 30 min in the GC injector following the manufacturer’s recommendations, was then used. Although equilibrium concentrations on the fibre were reached after approximately 20 min, extractions continued for 30 min to fit conveniently into the analysis cycle of the GC-MS. To prevent carry-over of (*E*,*E*)-α-farnesene from one analysis to the next due to adsorption on container walls, containers were thoroughly washed between analyses. Carry-over on the fibre was eliminated, as reanalysis of fibres resulted in blank chromatograms.

The SPME-coated fibre containing the headspace volatile compounds was introduced into the GC injection port at 250 °C and kept for 5 min for thermal desorption. The injection port, lined with a 0.75 mm (i.d.) splitless glass liner, utilized splitless injections for 5 min. Separation was achieved using an Optima FFAP column (30 m × 0.25 mm, 0.25 μm film thickness) with a helium flow rate of 1 mL/min. The oven temperature program was set at 40 °C for 5 min, increased to 155 °C at 5 °C/min, then ramped up to 300 °C at 20 °C/min and held at that temperature for 1 min. The transfer line was heated to 250 °C. The mass spectrometer operated in electron impact mode (EI) at 70 eV, scanning the range 33–300 m/z in a full scan acquisition mode. The GC peak area data were used to estimate the relative content of each volatile compound and to monitor changes during storage. Identification of volatile compounds was accomplished by comparing GC retention times and mass spectra with those of pure standard compounds, when available. All mass spectra were also compared with the data system library (Wiley 6) and other published spectra. A series of n-alkanes (C7–C30) were analyzed under the same conditions, and Kovats retention indexes were calculated and compared to those in the NIST online library (https://webbook.nist.gov/chemistry/name-ser/, accessed on 12 April 2024). All measurements were performed on five different apples (replicates).

### 4.11. Statistical Analysis

Results were expressed as mean ± standard deviation and were analysed by a one-way ANOVA with Tukey’s post hoc test. Differences between average values were reported as significant at *p* = 0.05. Statistical analyses were performed using Statistica 13.3 (Statsoft Inc., Tulsa, OK, USA).

#### 4.11.1. Principal Component Analysis (PCA)

Principal component analysis (PCA) is an unsupervised multivariate statistical analysis, whose main objective is to reduce the dimensionality of a data set containing a high number of interrelated variables, thus retaining as much as possible the variability present in the original set of data variables. From these results, it should be possible to highlight several characteristics and correlate them to the chemical composition of the different apple samples analysed. PCA was performed using STATISTICA 10 (StatSoft, Tulsa, OK, USA).

#### 4.11.2. Orthogonal Partial Least Squares (OPLS-DA)—Discriminant Analysis

Partial least squares-discriminant analysis (PLS-DA) is a regression method commonly used in multivariate statistics, to establish the relationship between 2 data information sets, referred to as X being the apples volatilome, and Y, a binary vector value. Orthogonal PLS-DA (OPLS-DA) is a modification of PLS-DA, which separates the systematic variation in X into 2 parts; one is linearly related to Y (tp) and the other is orthogonal to Y (to).The quality of the OPLS-DA model is evaluated by the goodness-of-fit parameter (R2X), the proportion of the variance of the response variable that is explained by the model (R2Y), and the predictive ability parameter (Q2), which is calculated by a 7-round internal cross-validation of the data. The parameters R2X and R2Y represent the fraction of the variance of matrix X and matrix Y, respectively, and Q2 represents the predictive accuracy of the model. R2X, R2Y and Q2 values close to 1 indicate an excellent OPLS-DA model, and values higher than 0.5 indicate an OPLS-DA model of good quality [107]. Additionally, the models obtained were also evaluated by randomly changing the order of the Y-observations (permutation) without changing the X values (999 permutations) to assess the risk that the obtained OPLS-DA model was spurious; i.e., the model obtained has a good fit for the training set but does not has a good prediction of Y new observations and the goodness of fit of the original model (R2 and Q2) were compared to the new models obtained by permutation. Analyses were performed using SIMCA 14 (Sartorius, Göttingen, Germany).

## 5. Conclusions

Storage conditions, namely normal atmosphere, controlled atmosphere, and 1-MCP treatment before controlled atmosphere storage, significantly affect the texture, colour, chemical composition and volatilome of ‘Golden Delicious’ and ‘Red Delicious’ apples. Apples stored under normal atmosphere and controlled atmosphere maintain firmness, while 1-MCP treatment before controlled atmosphere storage increases firmness in both apple cultivars. Treatment with 1-MCP alters the colour of apples, resulting in significant changes in chromaticity and luminosity. Storage conditions impact the total soluble sugar content, with normal storage causing the greatest decrease in glucose, sucrose and fructose levels, leading to changes in sweetness index and glycemic load. 1-MCP treatment leads to a more significant decrease in sucrose levels and an increase in glucose levels compared to controlled atmosphere storage alone. Only 1-MCP-treated apples show a significant decrease in malic acid content for both cultivars. Changes in malic acid and total soluble sugars, along with a decrease in total titratable acidity, will affect the taste of the apples after storage. All storage conditions result in significant changes in the abundance and composition of the volatilome, with differences observed between ‘Golden Delicious’ and ‘Red Delicious’ apples. While 1-MCP effectively reduces the abundance of (*E*,*E*)-α-farnesene in ‘Golden Delicious’ apples, its impact on ‘Red Delicious’ apples is more limited. Regardless of the cultivar, the presence of 1-MCP, normal atmosphere, or controlled atmosphere results in lower levels of certain critical volatile organic compounds (VOCs), such as 2-methylbutyl acetate, butyl acetate and hexyl acetate. The effectiveness of 1-MCP varies depending on the cultivar, with ‘Golden Delicious’ showing a better response than ‘Red Delicious’.

## Figures and Tables

**Table 1:** Firmness, total soluble solids (TSSs), titratable acidity (TA), pH and chromatic characteristics in ‘Golden Delicious’ (GD) and ‘Red Delicious’ (RD) apple varieties at harvest (H), normal atmosphere storage (NA), controlled atmosphere storage (CA) and post-harvest treatment with 1-methylcyclopropene (1-MCP).

Apple Variety	Storage	Firmness (N)	TSS (%)	TA (g/100 g Malic Acid)	pH	Luminosity (L)	Cromaticity (C)	Hue (h°)
	H	27.8 ± 6.4 ^1 b 2^	13.9 ± 0.6 ^a^	7.9 ± 1.2 ^a^	3.1 ± 0.2 ^d^	74.1 ± 2.1 ^a^	47.6 ± 2.5 ^b^	123.2 ± 0.3 ^a^
	NA	26.5 ± 1.9 ^b^	13.0 ± 0.9 ^a^	3.8 ± 0.5 ^c^	4.0 ± 0.1 ^a^			
GD	CA	28.5 ± 4.8 ^b^	12.8 ± 0.5 ^a^	2.7 ± 0.5 ^d^	3.8 ± 0.1 ^b^	73.9 ± 2.4 ^a^	54.2 ± 2.1 ^a^	122.7 ± 0.1 ^a^
	1-MCP	35.5 ± 5.4 ^a^	13.2 ± 0.7 ^a^	5.7 ± 0.8 ^b^	3.4 ± 0.1 ^c^	71 ± 2.7 ^b^	49.3 ± 2.6 ^b^	122.8 ± 0.2 ^a^
	H	35.3 ± 3.8 ^b^	13.2 ± 0.4 ^b^	4.5 ± 0.4 ^a^	3.9 ± 0.1 ^b^	35.4 ± 6.4 ^b^	39.2 ± 3.8 ^a^	28.4 ± 6.6 ^a^
	NA	33.6 ± 2.8 ^b^	14.3 ± 0.6 ^ab^	3.1 ± 0.5 ^b^	4.2 ± 0.1 ^a^			
RD	CA	32.7 ± 4.5 ^b^	15.3 ± 0.9 ^a^	3.0 ± 0.4 ^b^	3.9 ± 0.1 ^b^	35.7 ± 4.6 ^b^	34.2 ± 4.0 ^c^	28.9 ± 5.7 ^a^
	1-MCP	43.0 ± 6.6 ^a^	13.1 ± 0.6 ^b^	3.5 ± 0.4 ^b^	3.8 ± 0.1 ^b^	38.4 ± 6.2 ^a^	34.8 ± 5.3 ^b^	27.4 ± 5.3 ^a^

^1^ Values represent mean ± standard deviation (n = 5). ^2^ Samples that contain different letters are significantly different (*p* ≤ 0.05).

**Table 2:** Malic acid (g/kg) and soluble sugars (g/kg) in ‘Golden Delicious’ (GD) and ‘Red Delicious’ (RD) apple varieties at harvest (H) and during storage under normal atmosphere (NA), controlled atmosphere (CA) and after treatment with 1-MCP.

Apple Variety	Storage	Malic Acid	Sorbitol	Glucose	Sucrose	Frutose	Total Sugars	SI	GI	GL
GD	H	19.5 ± 2.36 ^1 ab 2^	0.9 ± 0.1 ^1 a 2^	9.1 ± 1.5 ^bc^	17.1 ± 2.0 ^a^	43.6 ± 3.2 ^ab^	70.7 ± 2.7 ^a^	100.2	45	4.7
	NA	22.6 ± 3.07 ^a^	1.4 ± 0.1 ^a^	6.7 ± 1.9 ^c^	11.3 ± 2.1 ^b^	28.6 ± 1.8 ^c^	48.0 ± 1.9 ^c^	64.9	46	3.2
	CA	17.1 ± 2.68 ^b^	1.0 ± 0.2 ^a^	11.2 ± 1.4 ^b^	10.8 ± 2.7 ^bc^	40.6 ± 3.8 ^b^	63.6 ± 3.1 ^b^	88.2	46	4.3
	1-MCP	13.5 ± 2.15 ^c^	1.1 ± 0.2 ^a^	14.4 ± 2.3 ^a^	8.1 ± 2.0 ^c^	46.5 ± 2.9 ^a^	70.1 ± 2.6 ^a^	98.0	46	4.8
RD	H	12.3 ± 1.74 ^a^	2.0 ± 1.2 ^a^	15.9 ± 3.6 ^b^	15.7 ± 3.1 ^a^	43.3 ± 2.5 ^a^	76.9 ± 2.8 ^a^	101.2	50	5.7
	NA	12.8 ± 2.29 ^a^	1.9 ± 0.7 ^a^	11.0 ± 1.4 ^c^	14.9 ± 2.6 ^a^	28.9 ± 2.3 ^c^	56.7 ± 2.1 ^c^	72.3	52	4.3
	CA	10.1 ± 1.66 ^ab^	2.3 ± 0.4 ^a^	18.9 ± 1.0 ^a^	6.3 ± 1.2 ^b^	41.4 ± 2.8 ^a^	68.9 ± 2.1 ^b^	90.0	52	5.1
	1-MCP	8.67 ± 2.68 ^b^	1.5 ± 0.5 ^a^	19.4 ± 3.1 ^a^	3.1 ± 1.2 ^c^	37.1 ± 3.7 ^b^	61.1 ± 3.3 ^c^	80.7	53	4.8

^1^ Values represent mean ± standard deviation (n = 5). ^2^ Samples that contain different letters are significantly different (*p* ≤ 0.05). SI—sweetness index; GI—glycemic index; GL—glycemic load.

**Table 3:** Volatile composition (area × 10^7^) of ‘Golden Delicious’ and ‘Red Delicious’ varieties at harvest (H), normal atmosphere storage (NA), controlled atmosphere storage (CA) and after treatment with 1-MCP (1-MCP).

	Aroma Compounds	KI	ID ^a^	Aroma descriptor	‘Golden Delicious’				‘Red Delicious’			
					H	NA	CA	1-MCP	H	NA	CA	1-MCP
	Esters											
**23**	Ethyl butanoate	1058	A,B,C	oxidized apple, sweet					2.3 ± 1.4 ^a,1,2^	1.0 ± 0.6 ^a^	n.d. ^b^	n.d. ^b^
**24**	Propyl propionate	1059	B,C	sweet, fruity					0.2 ± 0.1 ^a^	n.d. ^b^	n.d. ^b^	n.d. ^b^
**25**	Ethyl 2-methylbutanoate	1063	A,B,C	Fruity					2.0 ± 1.4 ^a^	0.8 ± 0.7 ^a^	n.d. ^b^	n.d. ^b^
**1**	Butyl acetate	1082	A,B,C	fruity, apple	5.0 ± 1.6 ^a^	0.9 ± 0.1 ^b^	0.4 ± 0.3 ^c^	0.01 ± 0.01 ^d^	2.5 ± 0.9 ^a^	0.4 ± 0.1 ^b^	0.03 ± 0.01 ^c^	n.d. ^d^
**2**	2-Methylbutyl acetate	1134	A,B,C	overall aroma, characteristic apple, banana like	4.2 ± 2.0 ^a^	0.2 ± 0.1 ^b^	0.2 ± 0.1 ^b^	0.05 ± 0.02 ^c^	10.5 ± 2.8 ^a^	1.7 ± 0.5 ^b^	n.d. ^c^	n.d. ^c^
**3**	Butyl propionate	1137	B,C	Fuity	0.8 ± 0.5 ^a^	0.2 ± 0.1 ^b^	0.02 ± 0.01 ^c^	n.d. ^d^				
**26**	Propyl 2-methylbutanoate	1151	B,C	fruity, apple					3.8 ± 1.5 ^a^	0.3 ± 0.1 ^b^	n.d. ^c^	n.d. ^c^
**4**	Pentyl Acetate	1168	A,B,C	fruity, apple, banana-like	1.0 ± 0.2 ^a^	0.09 ± 0.01 ^b^	0.04 ± 0.02 ^c^	n.d. ^d^				
**5**	Butyl butanoate	1221	A,B,C	rotten apple, cheesy	6.7 ± 1.0 ^a^	1.2 ± 0.3 ^b^	0.4 ± 0.3 ^c^	0.04 ± 0.01 ^d^	3.5 ± 0.8 ^a^	0.8 ± 0.3 ^b^	0.04 ± 0.02 ^c^	n.d. ^d^
**6**	Butyl 2-methylbutanoate	1239	A,B,C	fruity, apple	5.3 ± 1.0 ^a^	0.3 ± 0.1 ^b^	0.2 ± 0.1 ^b^	0.04 ± 0.02 ^c^	9.4 ± 3.4 ^a^	2.5 ± 1.3 ^a,b^	0.1 ± 0.1 ^a,b^	0.3 ± 0.3 ^b^
**7**	Hexyl acetate	1268	A,B,C	sweet fruity, apple	29.9 ± 7.2 ^a^	4.4 ± 0.8 ^b^	2.8 ± 1.4 ^b^	0.2 ± 0.1 ^c^	13.2 ± 3.6 ^a^	2.6 ± 0.6 ^a^	0.3 ± 0.1 ^a,b^	0.1 ± 0.2 ^b^
**8**	Pentyl butanoate	1305	A,B,C	banana	1.4 ± 0.1 ^a^	0.2 ± 0.1 ^b^	0.04 ± 0.02 ^c^	n.d. ^d^	0.8 ± 0.3 ^a^	0.1 ± 0.1 ^a,b^	n.d. ^c^	0.1 ± 0.1 ^b^
**27**	Propyl hexanoate	1313	B,C	Fruit					5.4 ± 1.9 ^a^	0.9 ± 0.4 ^a,b^	0.1 ± 0.1 ^a,b^	0.6 ± 0.6 ^b^
**9**	Pentyl 2-methylbutanoate	1332	B,C		1.0 ± 0.2 ^a^	0.02 ± 0.01 ^b^	n.d. ^d^	0.01 ± 0.01 ^c^	2.3 ± 0.8 ^a^	0.1 ± 0.1 ^a,b^	0.01 ± 0.01 ^a,b^	0.1 ± 0.1 ^b^
**11**	Hexyl 2-methylpropanoate	1339	B,C	sweet, green, fruity, apple-like	0.6 ± 0.3 ^a^	0.1 ± 0.0 ^b^	0.1 ± 0.0 ^b^	0.02 ± 0.01 ^c^				
**10**	Hexyl propionate	1354	B,C	fruity, apple	3.2 ± 2.2 ^a^	0.5 ± 0.1 ^b^	0.2 ± 0.1 ^c^	0.03 ± 0.01 ^d^	2.4 ± 0.5 ^a^	0.6 ± 0.2 ^b^	n.d. ^c^	n.d. ^c^
**28**	Pentyl pentanoate	1401	B,C	ripe, fruity, apple					1.8 ± 0.5 ^a^	0.2 ± 0.1 ^a^	n.d. ^b^	0.6 ± 0.6 ^a^
**12**	Butyl hexanoate	1406	B,C	green apple	28.3 ± 4.1 ^a^	5.8 ± 1.3 ^a^	1.7 ± 1.2 ^a,b^	0.1 ± 0.1 ^b^	32.0 ± 7.4 ^a^	6.2 ± 3.7 ^a^	0.8 ± 0.4 ^a^	3.3 ± 2.0 ^a^
**13**	Hexyl butanoate	1420	B,C	apple	20.6 ± 2.5 ^a^	5.4 ± 1.6 ^b^	2.7 ± 2.7 ^c^	0.2 ± 0.1 ^d^	3.2 ± 1.2 ^a^	3.9 ± 2.1 ^a^	0.3 ± 0.1 ^b^	0.5 ± 0.2 ^b^
**29**	Ethyl octanoate	1420	A,B,C	fruit, fat					0.4 ± 0.3 ^a^	0.4 ± 0.3 ^a^	n.d. ^b^	n.d. ^b^
**14**	Hexyl 2-methylbutanoate	1425	B,C	apple, grapefruit	59.4 ± 19.9 ^a^	3.8 ± 0.4 ^b^	4.5 ± 1.8 ^b^	1.0 ± 0.6 ^c^	75.9 ± 10.0 ^a^	13.9 ± 7.3 ^b^	1.4 ± 0.8 ^d^	5.2 ± 3.0 ^c^
**15**	Pentyl hexanoate	1501	B,C	sweet, green fruity, apple, fatty	0.7 ± 0.2 ^a^	0.5 ± 0.1 ^a^	0.2 ± 0.0 ^b^	0.1 ± 0.0 ^c^	3.7 ± 1.2 ^a^	0.8 ± 0.4 ^b,c^	0.4 ± 0.3 ^c^	2.0 ± 1.1 ^a,b^
**31**	Propyl octanoate	1510	B,C	coconut, cocoa, fatty					0.5 ± 0.1 ^a^	0.2 ± 0.1 ^a^	0.01 ± 0.01 ^b^	0.3 ± 0.2 ^a^
**16**	Butyl heptanoate	1514	B,C	Green, fruity, licorice, grassy	3.5 ± 0.2 ^a^	n.d. ^b^	n.d. ^b^	n.d. ^b^				
**17**	Hexyl hexanoate	1599	B,C	apple	34.7 ± 4.1 ^a^	11.9 ± 1.5 ^b^	5.2 ± 2.5 ^c^	0.9 ± 0.5 ^d^	30.8 ± 8.3 ^a^	13.3 ± 3.5 ^b^	1.9 ± 0.6 ^d^	5.8 ± 3.2 ^c^
**18**	Butyl octanoate	1619	B,C	Fruit	6.0 ± 0.9 ^a^	1.3 ± 0.5 ^b^	0.2 ± 0.1 ^c^	n.d. ^d^	2.0 ± 0.4 ^a^	1.6 ± 0.7 ^a^	0.2 ± 0.2 ^b^	0.9 ± 0.5 ^a^
**22**	Hexyl octanoate	1793	B,C	herb, green, oil	1.1 ± 0.2 ^a^	0.3 ± 0.1 ^b^	0.1 ± 0.0 ^c^	0.02 ± 0.01 ^d^	0.5 ± 0.1 ^b^	1.0 ± 0.4 ^a^	0.1 ± 0.01 ^c^	0.2 ± 0.1 ^b^
	Subtotal				213 ± 42 ^a^	37 ± 2 ^b^	19 ± 9 ^c^	2.7 ± 1.3 ^d^	209 ± 41 ^a^	53 ± 15 ^b^	5.7 ± 2.6 ^d^	22 ± 9 ^c^
	Subtotal (%)				57	48	33	11	52	53	6.3	14
	Phenols											
**19**	1-Methoxy-4-(2-propen-1-yl)-benzene (Estragol)	1685	B,C	licorice, anise	1.5 ± 0.5 ^a^	0.2 ± 0.1 ^b^	0.1 ± 0.1 ^c^	0.03 ± 0.01 ^c^	1.1 ± 0.3 ^a^	0.5 ± 0.1 ^b^	n.d. ^c^	n.d. ^c^
	Subtotal				1.5 ± 0.5 ^a^	0.2 ± 0.1 ^b^	0.1 ± 0.1 ^c^	0.03 ± 0.01 ^c^	1.1 ± 0.3 ^a^	0.5 ± 0.1 ^b^	n.d. ^c^	n.d. ^c^
	Subtotal (%)				0.4	0.3	0.1	0.1	0.3	0.5	0	0
	Terpenoids											
**20**	*trans*-α-Bergamotene	1592	B,C	fruity, bergamote	0.8 ± 0.2 ^a^	0.2 ± 0.0 ^b^	0.1 ± 0.1 ^b^	0.1 ± 0.0 ^b^	0.9 ± 0.1 ^a^	0.2 ± 0.1 ^c^	0.4 ± 0.1 ^b^	0.6 ± 0.2 ^a^
**21**	(*E*,*E*)-α-Farnesene	1757	A,B,C	green, floral, herbal, citrus	158 ± 17 ^a^	39.1 ± 5.1 ^b^	38.9 ± 13.6 ^b^	21.1 ± 8.2 ^c^	191 ± 26 ^a^	46.1 ± 10.8 ^d^	83.9 ± 16.1 ^c^	134 ± 23 ^b^
	Subtotal				159 ± 17 ^a^	39. ± 5 ^b^	39 ± 14 ^b^	21. ± 8 ^c^	191 ± 26 ^a^	46.3 ± 10.8 ^d^	84.3 ± 16.2 ^c^	134 ± 23 ^b^
	Subtotal (%)				42	51	67	88	48	46	94	86
	Total				373 ± 35 ^a^	77 ± 7 ^b^	58 ± 22 ^b^	24 ± 9 ^c^	402 ± 62 ^a^	100 ± 20 ^c^	90 ± 17 ^c^	156 ± 30 ^b^

^1^ Values represent mean ± standard deviation (%) (n = 5). ^2^ Samples that contain different letters are significantly different (*p* ≤ 0.05). The reliability of the identification or structural proposal is indicated by the following: A-mass spectrum and retention time consistent with those of an authentic standard; B-structural proposals given on the basis of mass spectral data (Wiley 6); C-mass spectrum consistent with spectra found in literature. Not detected (n.d.).

## Data Availability

Data will be made available on request.

## Supplementary Materials

The following supporting information can be downloaded at: https://www.mdpi.com/article/10.3390/molecules29132954/s1, Figure S1: Chromatograms of the volatile composition of Golden Delicious variety at harvest (A), normal atmosphere storage (B), controlled atmosphere storage (C), and after treatment with 1-MCP (D) (1-22 identification in Table 3); Figure S2: Chromatograms of the volatile composition of Red Delicious variety at harvest (A), normal atmosphere storage (B), controlled atmosphere storage (C), and after treatment with 1-MCP (D) (23–31 identification in Table 3).
