# Supplementary material for: Effect of Storage Conditions on the Volatilome, Biochemical Composition and Quality of Golden Delicious and Red Delicious Apple (Malus domestica) Varieties
Source: Molecules. 2024 Jun 21;29(13):2954. doi: 10.3390/molecules29132954 (PMC11243091; doi:10.3390/molecules29132954)
Supplement: Supplementary file 1 [file molecules-29-02954-s001.zip › molecules-3049315-supplementary.pdf]

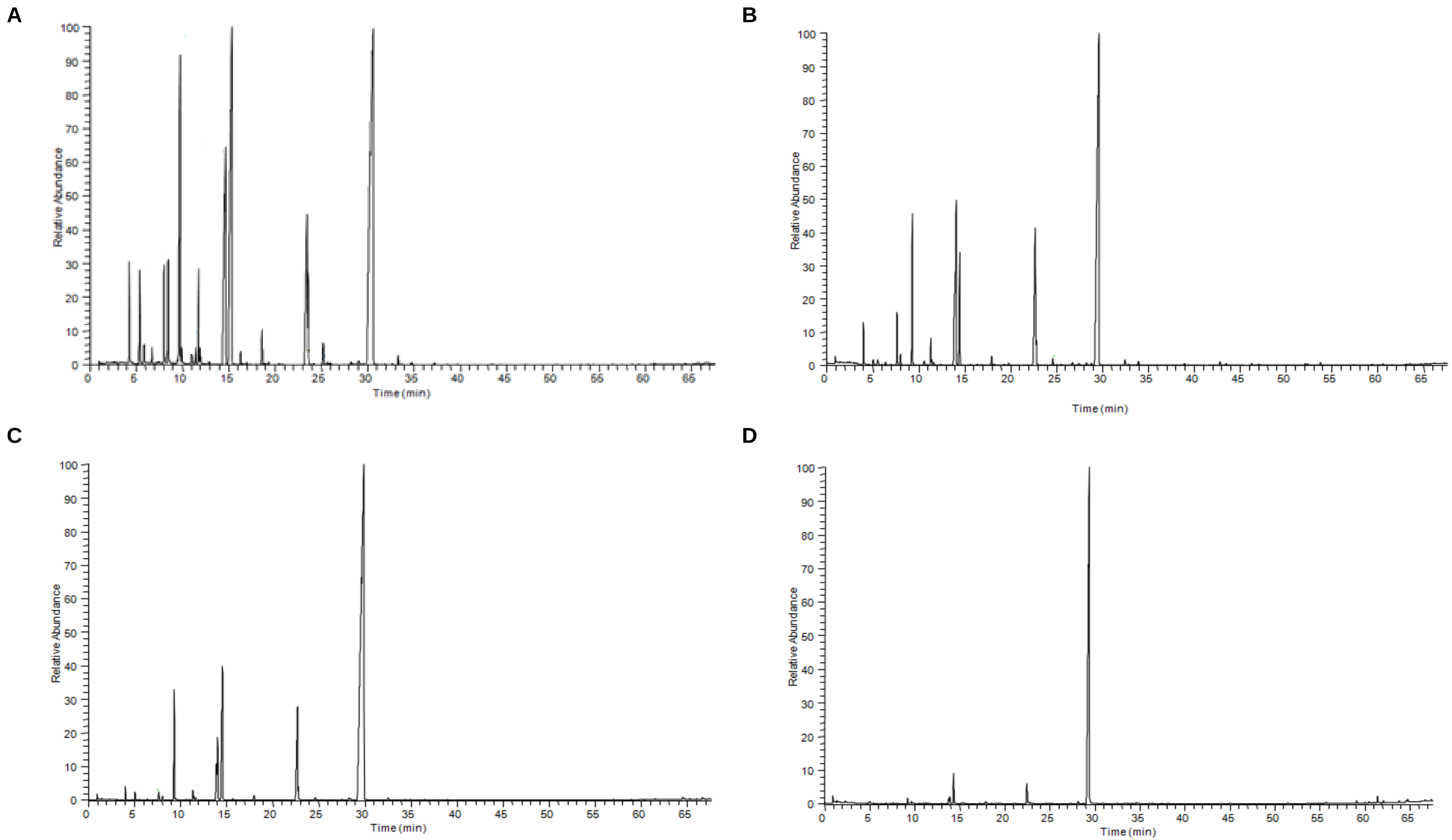

Figure S1. Chromatograms of the volatile composition of Golden Delicious variety at harvest (A), normal atmosphere storage (B), controlled atmosphere storage (C), and after treatment with 1-MCP (D) (1-22 identification in Table 3).

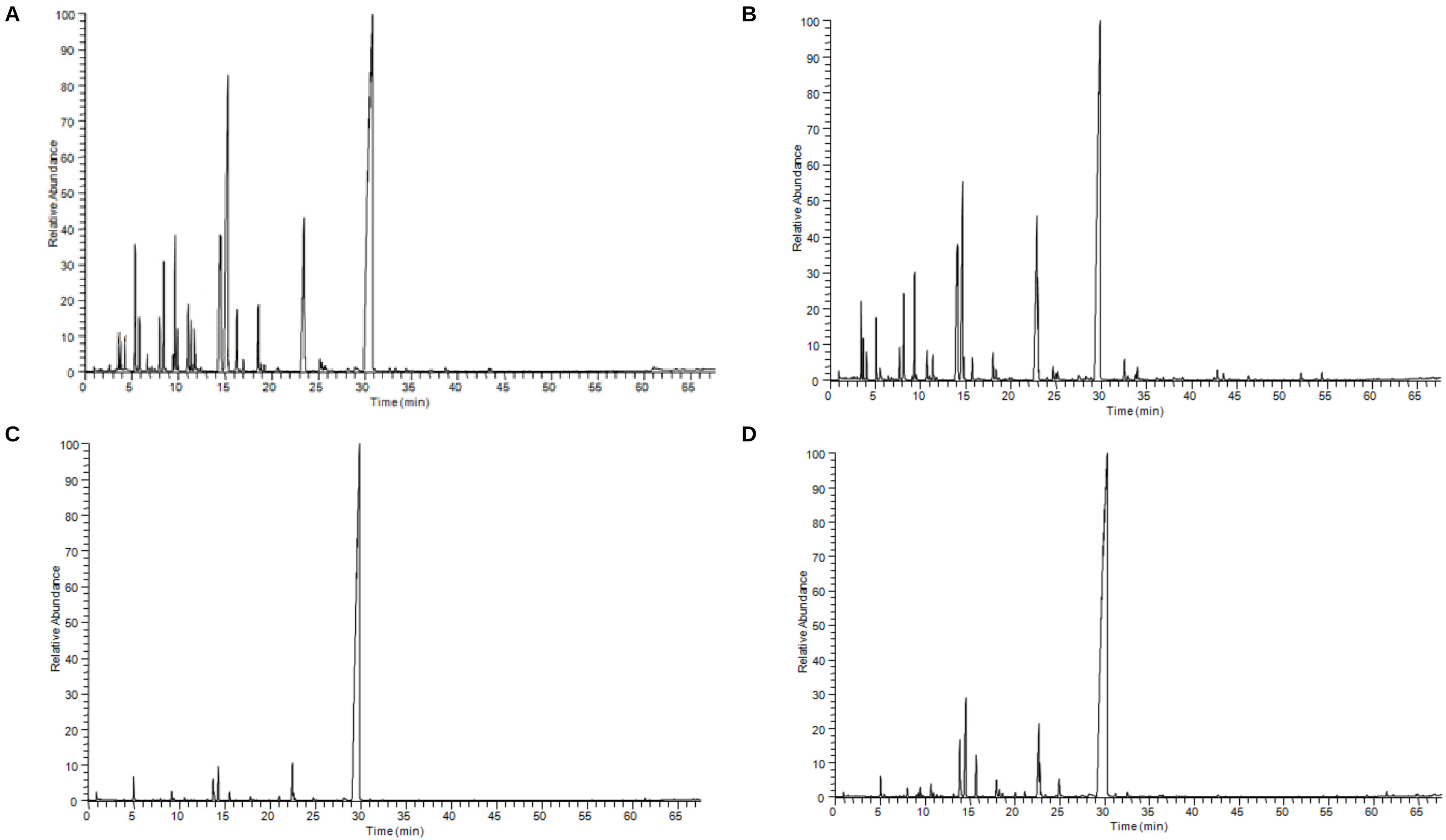

Figure S2. Chromatograms of the volatile composition of *Red Delicious* variety at harvest (A), normal atmosphere storage (B), controlled atmosphere storage (C), and after treatment with 1-MCP (D) (23-31 identification in Table 2).
